# Evidence for individual discrimination and numerical assessment in collective antipredator behaviour in wild jackdaws (*Corvus monedula*)

**DOI:** 10.1098/rsbl.2019.0380

**Published:** 2019-10-02

**Authors:** Jenny R. Coomes, Guillam E. McIvor, Alex Thornton

**Affiliations:** 1 Centre for Ecology and Conservation, University of Exeter, Penryn Campus, Penryn, Cornwall TR10 9FE, UK; 2 School of BEES, University College Cork, North Mall, Cork T23 N73K, Republic of Ireland

**Keywords:** numerical assessment, collective behaviour, antipredator, individual discrimination, *Corvus monedula*

## Abstract

Collective responses to threats occur throughout the animal kingdom but little is known about the cognitive processes underpinning them. Antipredator mobbing is one such response. Approaching a predator may be highly risky, but the individual risk declines and the likelihood of repelling the predator increases in larger mobbing groups. The ability to appraise the number of conspecifics involved in a mobbing event could therefore facilitate strategic decisions about whether to join. Mobs are commonly initiated by recruitment calls, which may provide valuable information to guide decision-making. We tested whether the number of wild jackdaws responding to recruitment calls was influenced by the number of callers. As predicted, playbacks simulating three or five callers tended to recruit more individuals than playbacks of one caller. Recruitment also substantially increased if recruits themselves produced calls. These results suggest that jackdaws use individual vocal discrimination to assess the number of conspecifics involved in initiating mobbing events, and use this information to guide their responses. Our results show support for the use of numerical assessment in antipredator mobbing responses and highlight the need for a greater understanding of the cognitive processes involved in collective behaviour.

## 1. Introduction

In many animal species, individuals come together to repel external threats. One such collective behaviour is mobbing: the joint harassment of a predator to drive it from the area. Mobbing is a collective action problem because it provides a collective benefit but entails substantial individual risk [1]. Mobbing events are commonly initiated by recruitment calls [2,3] and an individual's decision to join may depend on the information contained within these calls. Given that the risk to each individual is expected to decrease and the likelihood of driving away the predator rises in larger mobbing groups [4,5], the ability to appraise the number of individuals in a mob prior to joining may be highly beneficial.

Aspects of numerical assessment (i.e. assessment of relative natural numbers; rather than simply detecting the presence or the absence of stimuli) have been described in many animal species [6–8] and are thought to involve specialized neural and cognitive processes [9]. The great majority of research has been conducted in captivity, but the ability to assess numerosities is widely assumed to have ecological significance, promoting survival and reproduction [10]. Field studies are therefore vital to determine the role of numerical assessments in ecologically relevant contexts [6]. For instance, McComb *et al.* [11] used playbacks of roaring female lions to examine the role of numerical assessment during inter-group conflict, finding that prides were less likely to approach their opponents when they were outnumbered. In territorial conflicts, where individuals face substantial costs if they lose, assessing the number of intruders may be vital in deciding whether to defend the territory or back down [6,12,13]. Collective antipredator responses involve similar individual risks and collective benefits, but little is known about the role of numerical assessment in this context (but see [14–16]).

We tested whether the magnitude of collective mobbing responses in wild jackdaws (*Corvus monedula*) is influenced by the number of individuals producing antipredator recruitment calls. Jackdaws are small, colony-breeding corvids that use individually distinctive alarm calls (known as scold calls) to recruit conspecifics to mob predators [3]. Recruits can be drawn in from a wide area and may not initially see the predator, so these calls provide important information for individuals to decide whether to abandon their current activity to join the mob. Jackdaws are known to show larger group-level responses to the calls of familiar colony members than strangers [3], but whether they discriminate between the calls of different individual colony members remains unclear. Individual vocal discrimination may potentially allow listeners to determine the number of callers attempting to recruit assistance for mobbing. We used playbacks simulating calling by one, three or five jackdaws (while keeping the rate and total number of calls constant) to test the prediction that a larger number of callers would recruit more individuals.

## 2. Material and methods

Sound recordings and playbacks were conducted in Cornwall, UK at two nest-box colonies, Y (N 50°11′25.98″, W 5°10′49.00″) and Z (N 50°11′55.37″, W 5°10′7.48″) from April to June 2016. The jackdaws used in this experiment were all free-living adults, most of which had been colour ringed for individual identification.

### (a) Playback track creation

We recorded scold calls from male nest-box users (nine at colony Y and 12 at colony Z) that would be familiar to conspecifics within their breeding colony. To record calls, we approached the focal nest-box to elicit a scolding response (details in electronic supplementary material). Playback tracks were designed to simulate calling by one (group size 1: GS1 treatment), three (GS3) or five (GS5) individuals. This reflects numbers of scolding birds that commonly initiate natural mobbing events, while keeping the increase in the number of birds constant between treatments.

We used Audacity® software [17] to extract discrete scold calls from our recordings, normalize call amplitude [3] and create playback tracks. All tracks followed the same structure, which comprised 15 calls: three bouts of five separated by 10 s with 2 s between calls in a bout to ensure the same rate of calling. Tracks comprising a single caller (GS1) used 15 different calls for that one individual (electronic supplementary material, figure S1a). Tracks comprising multiple callers used, in a random order, five different calls from three individuals (GS3; electronic supplementary material, figure S1b) or three different calls from five individuals (GS5; electronic supplementary material, figure S1c).

While natural scolding events sometimes show some overlap between the calls of different callers, our playback track design allowed us to ensure that calls by different individuals were audible to receivers. Individual callers were randomly assigned to treatments, ensuring that all multiple-caller tracks had different combinations of individuals. All playback tracks used in the experiment were unique (see the electronic supplementary material, tables S1 and S2 for lists of individuals used and their locations for playbacks).

### (b) Playbacks

Experiments were performed during the nesting period (April–June) using a remote controlled loudspeaker (FoxPro Fury 2) less than 20 m away from an occupied nest-box. Playback treatments (GS1, GS3 and GS5) were conducted in a random order between 7.00 and 18.00 at eight different locations per colony (locations within a colony were separated by at least 60 m), giving a total sample size of 48 playbacks across the two colonies (electronic supplementary material, tables S1 and S2). An HD Panasonic video camera (HC-X900) was set up 30–50 m away to record the birds' responses, and after allowing time for nearby jackdaws to return to natural behaviour, we played the chosen playback track (see the electronic supplementary material for further details).

We used the Behavioural Observation Research Interactive Software (BORIS) [18] to transcribe the video records, noting the number of recruits in total and whether the recruits themselves scolded. A recruit was classified as any jackdaw that moved to within 20 m of the speaker and/or circled above the speaker. Twenty per cent of videos were analysed by a second coder who was blind to treatment, showing strong agreement between raters (intraclass correlation coefficient: ICC = 0.998; 95% confidence interval: 0.995–0.999; *p* < 0.001).

### (c) Analysis

Data were analysed in R version 3.4.1 [19]. The *lme4* package [20] was used to create a generalized linear mixed model with a Poisson error distribution and a log link function that contained all the possible explanatory variables. The response variable was the number of recruits, and treatment (GS1, GS3 or GS5), trial number (1st, 2nd, 3rd playback at location), time of day (continuous) and date were fitted as explanatory terms. As scolding by recruits could serve to amplify playback stimuli [3] and wind could attenuate the broadcast sound, we fitted responsive scolding (yes/no) and wind speed (obtained from Carnkie Weather Station: carnkieweather.co.uk) as additional explanatory terms. We included colony (Y/Z) as a random effect, with the exact location where the playback was performed nested within this.

We used the *dredge* function in the *MuMIn* package [21] to determine which of the 11 models derived from our global model received the most support, with treatment and the presence/absence of responsive scolding being fixed terms in all models, as these were integral to our experimental predictions. The minimum number of parameters allowed in the model was therefore 2, and the maximum was set at 4 to avoid over parametrization. No interaction effects were tested. Owing to overdispersion in our data, we performed model selection with models ranked by QAICc (quasi-AIC corrected for small sample sizes [22]). Application of the nesting rule [23] formed the top set of models, ensuring that more complex versions of the model with the lowest QAICc value were not retained. Only models with a ΔQAICc < 6 were retained in the final group for calculation of model weights. A quasi-correction was applied to all model summaries to make the reported results more conservative. Given that responsive scolding may mask the effects of experimental treatment, we also ran tests using only trials without responsive scolding (*n* = 35). Model fit was assessed by using standard residual plot techniques and Cook's distances were calculated to identify data points that were potentially highly influential (Cook's distance > 1). When these occurred, the models were rerun with these data points excluded; in all cases the results remained consistent. Reported model *R*^2^-values indicate the proportion of variation explained by the models with (conditional) and without (marginal) the random effects being taken into account. To test whether the presence/absence of specific individual callers in the playback had a biasing effect on the number of birds that recruited, we re-ran our reported models using the package *MCMCglmm*, including a multi-membership random term for each individual caller (see the electronic supplementary material).

## 3. Results

**Figure 1.**
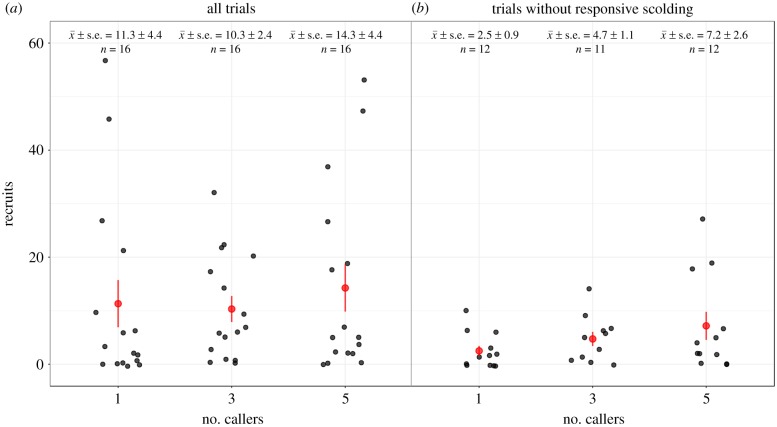
Number of recruits in the response to playbacks for one, three and five callers for (*a*) the full dataset and (*b*) the subset of data without responsive scolding by recruits. Datapoints were randomly shifted horizontally to avoid overlap, using the *jitter* function in ggplot2. Black points are raw data from each playback trial; the mean and standard error for each number of callers are shown in red.

Our analysis returned three candidate models within the top set following application of the nesting rule (table 1), with *model 3* receiving the greatest level of support (table 2; see electronic supplementary material, table S3 for summaries of the models receiving less support). The occurrence of responsive scolding by recruits increased the number of individuals recruited to the playbacks (GLMM, *b* (s.e.) = 1.780 (0.254), *z* = 7.01, *p* < 0.001, figure 1 and table 2). In addition to this effect, the GLMM also showed an increase in the number of recruits as the number of callers in the playback track increased but this only identified a clear difference between GS1 and GS5 (*b* (s.e.) = 0.751 (0.277), *z* = 2.71 *p* = 0.007, table 2), but not between GS1 versus GS3 (*b* (s.e.) = 0.487 (0.298), *z* = 1.63, *p* = 0.102, figure 1*a* and table 2). As confirmation, we also analysed the subset of the dataset in which no responsive scolding occurred (*n* = 35) and found an increased effect of treatment, indicating that the presence of responsive scolding masked the effect of our experimental treatments (GS1 versus GS3, *b* (s.e.) = 1.004 (0.417), *z* = 2.41, *p* = 0.016; GS1 versus GS5, *b* (s.e.) = 1.499 (0.377), *z* = 3.97, *p* < 0.001; post-hoc comparison of GS3 versus GS5 *b* (s.e.) = 0.510 (0.294), *z* = 1.73, *p* = 0.083, figure 1*b*; electronic supplementary material, table S4). Our main analysis also identified a potential habituation effect, with recruits declining as trial number increased (*b* (s.e.) = −0.423 (0.149), *z* = −2.83, *p* = 0.005, table 2). Similarly, *model 2* identified a weak effect of date, with fewer recruits as the breeding season progressed (table 1; electronic supplementary material, table S3; see supplementary discussion). This effect was not robust once the quasi-correction to model outputs had been applied (*b* (s.e.) = −0.022 (0.014), *z* = −1.54, *p* = 0.12). Our analyses found no influence of wind speed or time of day. Additional post-hoc *MCMCglmm* analyses showed that including individual caller(s) within a multi-membership random term had no appreciable effect on model findings (electronic supplementary material, table S5).

**Table 1.** Model selection summary for the analysis identifying the predictors of the number of jackdaws that recruit to mobs after playbacks with one, three and five individuals calling. The models presented are those retained after application of the nesting rule [23] ranked by QAICc, with the top set highlighted in grey and the best-supported model in italics. The model *R*^2^_m_ and *R*^2^_c_ refer to the marginal and conditional *R*^2^ of each model, respectively, with the *R*^2^_c_ including the variation explained by both the fixed factors and random effects, while the *R*^2^_m_ reports the level of variation explained by the fixed factors only.

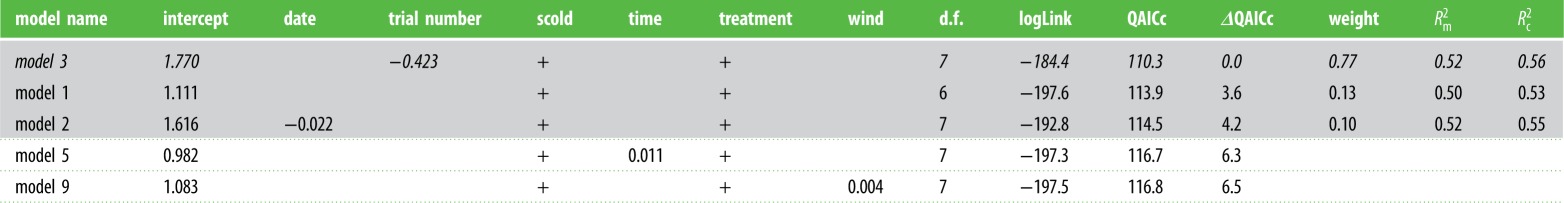

**Table 2.** Summary for the best-supported model in table 1 (*model 3*). The variance (s.d.) attributed to the nested random term colony/location is 0.433 (0.658) and to colony is 0.00 (0.00).

model 3				
variable	estimate	s.e.	*z*-value	*p*-value
intercept	1.770	0.452	3.92	<0.001
trial number	−0.423	0.149	−2.83	0.005
responsive scolding				
no	0	0		
yes	1.780	0.254	7.01	<0.001
treatment				
GS1	0	0		
GS3	0.487	0.298	1.63	0.102
GS5	0.751	0.277	2.71	0.007

## 4. Discussion

Little is known about the cognitive processes involved in regulating contributions to collective actions. Here, we investigated whether the magnitude of collective responses to antipredator recruitment calls is influenced by the number of callers. As predicted, we found that more callers elicit more recruits. These results are consistent with the argument that jackdaws use individual vocal discrimination to assess the number of individuals calling, suggesting that numerical assessment plays a role in influencing antipredator mobbing responses.

The ability to evaluate the size of mobbing groups before deciding whether to join could provide a number of benefits. First, as the number of individuals involved in a mobbing event increases, the risk to a single individual decreases exponentially, assuming all individuals invest identically [16]. Second, a greater number of individuals is better able to drive away a predator quickly [24,25] so individuals in large groups need invest less time and energy in mobbing than if defending in a smaller group [26]. Finally, it is possible that a greater number of callers may indicate to potential recruits that the information about the predator is more reliable.

The decision to join a larger mob must be underpinned by cognitive processes for appraising group sizes. If decisions are based on information contained in recruitment calls, individual call discrimination may be necessary to determine the number of different individuals calling. In our experiment, the total number of calls and the rate of calling were the same across all playbacks, with the only difference being the number of individuals whose calls were represented. Thus, by demonstrating differential responses linked to the number of individuals calling, our results suggest that jackdaws discriminate between the scold calls of different, individual colony members, building on previous evidence for discrimination between familiar and unfamiliar individuals [3]. We can exclude the possibility that our results are an artefact of certain individuals having particularly influential calls because *MCMCglmm* analyses show that the presence or the absence of specific individuals' calls within playback tracks had no influence on recruitment. In principle, our results could arise if multi-caller tracks contain greater levels of acoustic variation *per se*, independent of the number of callers. However, given previous evidence that jackdaw vocalizations (including scold calls) exhibit individual variation that elicits differential responses [3,27–30], our findings are strongly consistent with the argument that individual call discrimination enables jackdaws to appraise the number of callers. While there is evidence for numerical assessment in captive corvids (e.g. [31,32]), with individuals discriminating between different numbers up to 30 [33], our work suggests that such abilities play an important function in guiding behaviour in the wild.

Although our findings suggest that jackdaws employ numerical assessment under natural conditions, they also indicate there may be limitations to this ability. While there was an overall effect of experimental treatment on the number of recruits, we did not find clear and compelling evidence that the number of recruits was higher in response to five versus three callers. A possible reason for this may be that there is little further benefit to the jackdaws in assessing the number of callers above three. Alternatively, there may be cognitive limitations that curb the ability to assess the number of callers above a certain threshold [11,34]. While laboratory studies show that some corvids can discriminate considerably larger numbers [33], the potential for error may be substantially larger under natural conditions where individuals’ attention is divided among numerous ecologically relevant stimuli. Moreover, in our experiment, the magnitude of differences in caller numbers may have limited the potential for discrimination between treatments. Discrimination between pairs of numbers typically follows Weber's Law, whereby the discriminability of two numbers is dependent on the ratio of the difference, rather than the absolute difference, between them [6,35]. In our study, the absolute difference between the number of callers was equal across treatments (an addition of two callers from one to three to five), but the ratio between one and three is larger, and thus may be easier to discriminate, than that between three and five. Finally, the occurrence of responsive scolding, which stimulated additional recruitment, may have further masked apparent differences between experimental treatments (indeed, analysis of trials where no responsive scolding occurred revealed stronger effects of treatment, with a post-hoc test showing weakly suggestive evidence for more recruits in response to five than to three callers). To determine the cognitive limits of jackdaw numerosity, future work could manipulate the ratios between the number of callers to test if there is a maximal limit of number of callers that jackdaws can identify. It will also be important to determine whether numerical assessments integrate social information such as the familiarity, sex or dominance status of callers.

In conclusion, our results suggest that numerical assessment is important to a wild animal when deciding to join a collective antipredator behaviour. We provide evidence that in wild jackdaws, individual call discrimination allows assessment of the number of callers recruiting to a mobbing event, and thus affects the magnitude of the group response. Our findings add to our growing understanding of the role of cognitive processes in the formation and maintenance of collective responses in nature [3,36,37].

## Supplementary Material

Supplementary information: Evidence for individual discrimination and numerical assessment in collective antipredator behaviour in wild jackdaws (Corvus monedula). Coomes JR, McIvor GE, Thornton A.

## Data accessibility

Data available from the Dryad Digital Repository: https://dx.doi.org/10.5061/dryad.006bn4k [38].
