# Supplementary material for: Evidence for individual discrimination and numerical assessment in collective antipredator behaviour in wild jackdaws (Corvus monedula)
Source: Biol Lett. 2019 Oct 2;15(10):20190380. doi: 10.1098/rsbl.2019.0380 (PMC6832194; doi:10.1098/rsbl.2019.0380)
Supplement: Supplementary information: Evidence for individual discrimination and numerical assessment in collective antipredator behaviour in wild jackdaws (Corvus monedula). Coomes JR, McIvor GE, Thornton A. [file rsbl20190380supp1.pdf]

Supplementary information: ***Evidence for individual discrimination and numerical assessment in collective antipredator behaviour in wild jackdaws (Corvus monedula)***

Coomes JR, McIvor GE, Thornton A

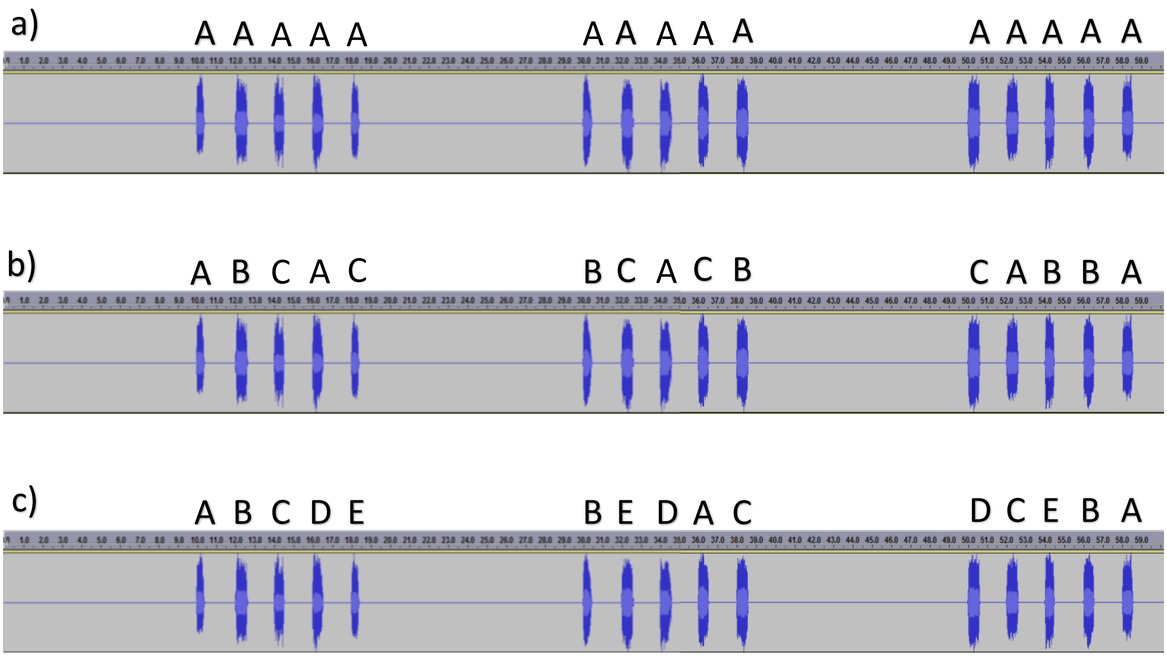

Figure S1: Playback tracks for the three treatments: a) GS1, b) GS3 and c) GS5. All playback tracks follow the same structure: 15 calls separated into three sets of five, each separated by 10 seconds. Calls within a set are separated by two seconds meaning a single track lasts 60 seconds. The letters above the calls represent different individual callers, providing examples of how the calls from the different individuals are spread throughout the track.

Table S1: The nest-box number of the different individuals used for each treatment at each location at colony Y. Locations S1 to S8 denote different places in the vicinity of the nest-box colony from which playbacks were broadcast. Different locations were separated by at least 65m.

Location	Treatment	Individuals used	Date
S1	GS1	Y29	04/05/2016
S1	GS3	Y11,04,07	15/04/2016
S1	GS5	Y07,19,29,01,11	02/05/2016
S2	GS1	Y10	14/04/2016
S2	GS3	Y06,04U,29	08/05/2016
S2	GS5	Y04U,11,19,06,10	03/05/2016
S3	GS1	Y07	15/04/2016
S3	GS3	Y07,11,01	12/04/2016
S3	GS5	Y29,06,11,04U,01	09/05/2016
S4	GS1	Y06	25/04/2016
S4	GS3	Y01,07,10	13/04/2016
S4	GS5	Y01,04,07,10,11	11/04/2016
S5	GS1	Y04U	06/05/2016
S5	GS3	Y04,10,11	12/04/2016
S5	GS5	Y11,07,01,10,04U	08/05/2016
S6	GS1	Y01	15/04/2016
S6	GS3	Y10,01,04	13/04/2016
S6	GS5	Y04,07,10,11,01	11/04/2016
S7	GS1	Y11	14/04/2016
S7	GS3	Y04U,29,10	06/05/2016
S7	GS5	Y19,04U,06,29,07	03/05/2016
S8	GS1	Y19	27/05/2016
S8	GS3	Y29,19,04U	26/05/2016
S8	GS5	Y01,29,04U,19,10	01/06/2016

Table S2: The nest-box number of the different individuals used for each treatment at each location at colony Z. Locations P1 to P8 denote different places in the vicinity of the nest-box colony from which playbacks were broadcast. Different locations were separated by at least 60m.

**Location**	**Treatment**	**Individuals used**	**Date**
P1	GS1	Z30	14/04/2016
P1	GS3	Z15,26,20	26/04/2016
P1	GS5	Z14,22,18,20,19	24/04/2016
P2	GS1	Z14	15/04/2016
P2	GS3	Z19,30,26	24/04/2016
P2	GS5	Z18,28,20,26,22	30/05/2016
P3	GS1	Z26	18/04/2016
P3	GS3	Z18,28,22	25/04/2016
P3	GS5	Z22,14,28,02,30	21/04/2016
P4	GS1	Z20	18/04/2016
P4	GS3	Z02,22,18	24/04/2016
P4	GS5	Z30,19,14,18,15	25/04/2016
P5	GS1	Z22	21/04/2016
P5	GS3	Z30,19,15	12/04/2016
P5	GS5	Z02,20,15,19,18	28/04/2016
P6	GS1	Z02	18/04/2016
P6	GS3	Z14,22,19	26/04/2016
P6	GS5	Z20,02,26,30,28	19/04/2016
P7	GS1	Z43	23/05/2016
P7	GS3	Z15,24,02	07/05/2016
P7	GS5	Z15,26,24,22,20	08/05/2016
P8	GS1	Z28	27/04/2016
P8	GS3	Z24,02,28	10/05/2016
P8	GS5	Z24,30,22,28,26	03/05/2016

Supplementary information for methods

Scold call recording

To minimise the effects of confounding variables, we used only the scold calls of male nest-box users that would be familiar to conspecifics within their breeding colony (nine males at colony Y and 12 at colony Z). Sample sizes were determined by the number of individuals for whom we could obtain high quality call exemplars. Calls were recorded using an Olympus LS-100 PCM recorder recording at 48.0Hz/16bit and a Sennheiser M67/K6 directional microphone with a Reinhardt windshield. All but one of the males recorded were colour-ringed for individual identification. For the remaining male (Y04U), his nest-box was known but he was not ringed so, as jackdaws are sexually monomorphic, we determined which bird of the pair was the male using behavioural observations (females incubate the nest while males provision their partner with food).

Approaching the focal nest-box to within 10m was sufficient to elicit scolding calls in the majority of cases. In five cases, climbing to within 1m of the nest-box was necessary to elicit scolding. Recordings collected with these two methods were randomly allocated to treatments (note that responses to playbacks of calls recorded using the two methods do not differ (1)).

Playback track creation

Playback tracks began with ten seconds of silence and the total track length was one minute. Tracks comprising a single caller (GS1) used 15 different calls for that one individual (Figure S1a). Tracks comprising multiple callers used, in a random order, five different calls from three individuals (GS3) (Figure S1b) or three different calls from five individuals (GS5) (Figure S1c). For nine of the 20 individuals recorded, the number of discrete scold calls available was limited, so calls had to be repeated within the playback track. For the 16 individuals that occurred in the GS1 tracks, eight had at least one call repeated (range: three calls having to be repeated three times, to only a single call having to be repeated once).

We note that, because of the number of discrete scold calls available in our recordings, a subset of the playback tracks contained call units that were repeated (see above). As it is theoretically possible that the repetition of call elements could influence responses, we ran an additional analysis on the subset of playback tracks that contained no repeated elements (n = 37). This analysis confirmed the strong effect of treatment, as in our main analysis (GLMM with quasi-correction for overdispersion: GS1 vs GS3, b (s.e.) = 1.018 (0.405), z = 2.52, p = 0.01; GS1 vs GS5, b (s.e.) = 1.228 (0.378), z = 3.25, p = 0.001).

Playback experiments

Experiments were performed at eight locations within each of the two colonies and as individuals move widely within the colony, this design ensured we did not always play any given individual's calls at the same specific location within the colony. Eight locations were chosen as this was the greatest number of repeats that could be done within the confines of the colonies, ensuring that the locations within each were a minimum of 60m apart. The average distance between locations at colony Y was 172.14m and between locations at colony Z was 155.71m. Only the calls of familiar colony members were played at each of the colonies i.e. individuals from colony Y only had their calls played at colony Y and the same for colony Z. To minimise the potential influence of birds recognising their own calls, we conducted playbacks as far as possible from the nest-box(es) of the individual(s) whose calls were being broadcast. (Note that given that jackdaw scold calls are monosyllabic and that sound attenuates through air and cranial bones, self-recognition of calls is highly unlikely; see (2) for further discussion).

As the presence of the researcher could cause disturbance we allowed time for the jackdaws to return to natural behaviour (mean = 3.5mins, min = 2mins, max = 12mins) before beginning each playback, i.e. for any scold calling and antipredator behaviour in the area to cease. No more than three treatments were done per colony per day and playbacks at the same colony were separated by at least four hours. At each location, the FoxPro speaker was always placed in precisely the same orientation and position, on a field boundary wall, 1-2m off the ground. The volume on the FoxPro remote control was set to 20, replicating the volume of a scold response from a jackdaw at the same distance, as measured using a Voltcraft SL-100 sound level meter. At each of the 16 locations, no track was played more than once meaning a total of 16 playback tracks were used for each treatment (see Table S1 and S2).

Supplementary information for results

Whole experiment:

The number of recruits to playbacks varied between 0 and 57 (mean ± s.e. = 11.95±2.20). For GS1, the average number of recruits was 11.31±4.41 (range: 0-57), for GS3 the average number of recruits was 10.31±2.43 (range: 0-32) and for GS5 the average number of recruits was 14.25±4.42 (range: 0-53).

Subset of dataset that did not including responsive scolding by recruits:

The number of recruits to playbacks where there was no responsive scolding varied between 0 and 27 (mean ± s.e. = 4.80±1.06). For GS1, the average number of recruits was 2.50±0.93 (range: 0-10), for GS3 the average number of recruits was 4.73±1.32 (range: 0-14) and for GS5 the average number of recruits was 7.17±2.61 (range: 0-27).

Table S3: Values from the 3 retained candidate GLMM models from the analysis that tested which variables predicted the number of birds that recruited in response to playbacks containing the scold calls of variable numbers of individuals. The three models reported were retained after application of the nesting rule, and had a ΔQAICc <6 of the model with the lowest QAICc value. The variance (s.d.) of the nested random effects of each model are given below in their respective summary table (also see Figure S2). *Model 3* (a) was the best supported model with a weight of 0.77. *Model 1* (b) had the second most support with a weight of 0.13, while *model 2* (c) had the lowest level of support with a weight of 0.10.

**a)** *model 3*				
Variable	Estimate	s.e.	z-value	*p*-value
Intercept	1.770	0.452	3.92	<0.001
Trial number	-0.423	0.149	-2.83	0.005
Responsive Scolding: No	0	0		
Yes	1.780	0.254	7.01	<0.001
Treatment: GS1	0	0		
GS3	0.487	0.298	1.63	0.102
GS5	0.751	0.277	2.71	0.007
colony/location = 0.433 (0.658)				
colony = 0.00 (0.00)				
**b)** *model 1*				
Variable	Estimate	s.e.	z-value	*p*-value
Intercept	1.111	0.422	2.64	0.008
Responsive Scolding: No	0	0		
Yes	2.061	0.255	8.07	<0.001
Treatment: GS1	0	0.000		
GS3	0.133	0.275	0.48	0.630
GS5	0.493	0.264	1.87	0.062
colony/location = 0.388 (0.623)				
colony = 0.00 (0.00)				
**c)** *model 2*				
Variable	Estimate	s.e.	z-value	*p*-value
Intercept	1.615	0.521	3.10	0.002
Date	-0.022	0.014	-1.54	0.12
Responsive Scolding: No	0	0		
Yes	1.894	0.267	7.10	<0.001
Treatment: GS1	0	0		
GS3	0.290	0.293	0.99	0.320
GS5	0.694	0.295	2.35	0.020
colony/location = 0.422 (0.650)				
colony = 0.00 (0.00)				

Table S4: Values of the GLMM model which tested the effects of number of callers in the playback (GS1 = 1, GS3 = 3, GS5 = 5) and trial number (i.e. the number of playbacks that had been conducted at that location), on the number of recruits, in the absence of responsive scolding. The variance (s.d.) of the nested random effect is given below the table. An additional post-hoc test showed only weak evidence for a slightly higher number of recruits for GS5 than GS3: *b* (s.e.) = 0.510 (0.294); z = 1.73, *p* = 0.083.

Variable		Estimate	s.e.	z-value	*p* -value
Intercept		1.479	0.570	2.59	0.01
Trial number		-0.528	0.199	-2.66	0.008
Treatment:	GS1	0	0		
	GS3	1.004	0.417	2.41	0.016
	GS5	1.499	0.377	3.97	<0.001
colony/location = 0.891 (0.944)					
colony = 0.00 (0.05)					

Potential effects of influential callers

In theory, apparent differences between treatment groups could arise as an artefact if the calls of certain individuals are particularly influential in recruiting conspecifics. To test whether the presence/absence of specific individual callers in the playback had a biasing effect on the number of birds that recruited, we used the package *MCMCglmm* to perform post-hoc tests which included a multi-membership random term for each individual caller (Bayesian analysis with 20,000 iterations). This multi-membership random term allows us to account for the effect of the presence or absence of each individual caller within each playback track (both single caller and multi-caller tracks), so highlighting whether individual callers have a strong influence on the number of recruits. These extra analyses showed that the variance for the multi-membership random term was not significantly different from zero, confirming that the effects of treatment (GS1, GS3, GS5) cannot be explained by certain callers eliciting greater responses (Table S5).

In addition to the evidence from MCMCglmm analyses, we note that in multiple-caller treatments, any effects of influential callers are likely to be attenuated by the inclusion of other individuals' calls: a GS3 or GS5 playback containing calls from an influential caller would also be likely to contain calls from less influential callers. Indeed, there is a great amount of variation in the levels of the response to playback tracks containing any given individual (Figure S3). For example, while Z14 elicited the

most recruits in GS1, the number of recruits to tracks containing Z14's calls varied widely in the other treatments (only 3 recruits in GS3 and from 0 – 27 in GS5).

Table S5: Summary table of the mean variance values returned for the individual random effect in the MCMCglmm post hoc tests, when a) the full data set was analysed, and b) when only trials where there was no responsive scolding was included. All models contained the same terms as those reported elsewhere in the manuscript, and were run for 20,000 iterations. Inclusion of the individual multi-membership random effect had little influence on the model values, and in no instance was the variance value significantly different from zero, indicating that there was no influence of the presence/absence of individual callers on the number of birds that recruited to the playback.

**a)** full			
	Mean	Lower 95% CI	Upper 95% CI
model 1	0.005	0.000	0.022
model 2	0.005	0.000	0.028
model 3	0.002	0.000	0.011
**b)** with no responsive scolding			
	Mean	Lower 95% CI	Upper 95% CI
model	0.002	0.000	0.001

Video clip

For an example of a response see the video clip which comprises a playback of five individuals calling. DOI: 10.6084/m9.figshare.8152958

Note on influential datapoints

In two of the analyses reported in the main text, post-model checks of Cook's distances revealed 3 data points that had the potential to have a biasing effect on our analysis. To investigate, we re-ran each analyses with the highlighted data point(s) removed. Our findings remained the same when these data points were excluded, which suggests our findings are robust and our analyses were not biased by extreme/outlying values.

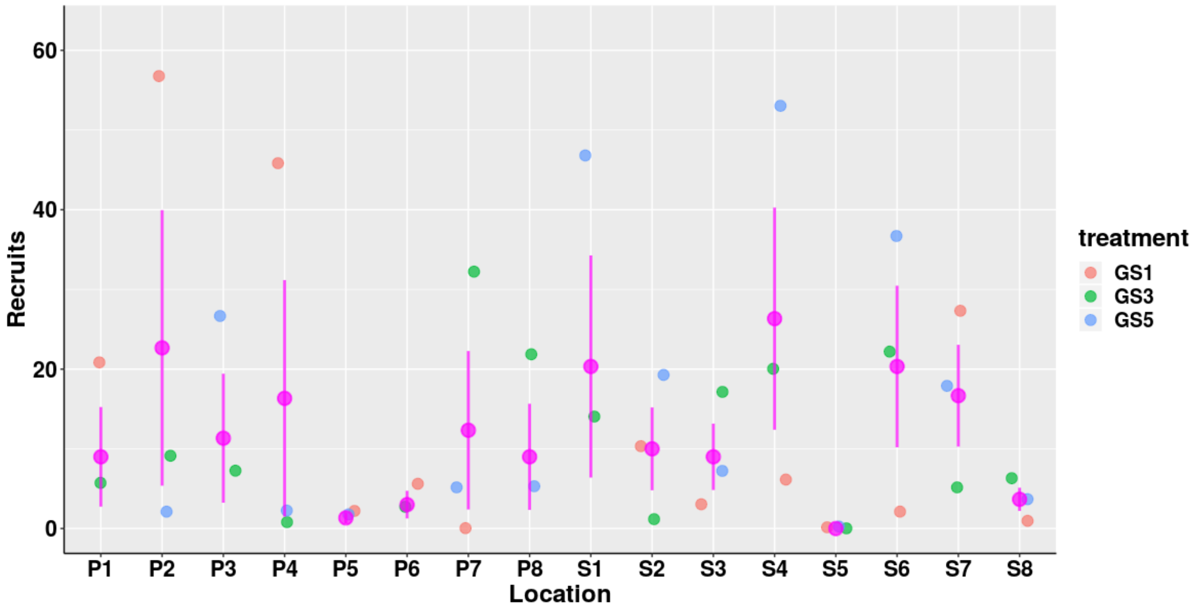

Figure S2: Plot displaying how the number of birds that recruited to playbacks varied at each of the 16 locations (P1-P8 at colony Z and S1-S8 at colony Y). Three playbacks were done at each location, one containing the calls of a single caller (GS1 – red dots), another with three calling males (GS3 – green dots), and the last with five calling males (GS5 – blue dots). The mean number of recruits for each location is denoted by the magenta point, with the error bars representing the standard error of the mean.

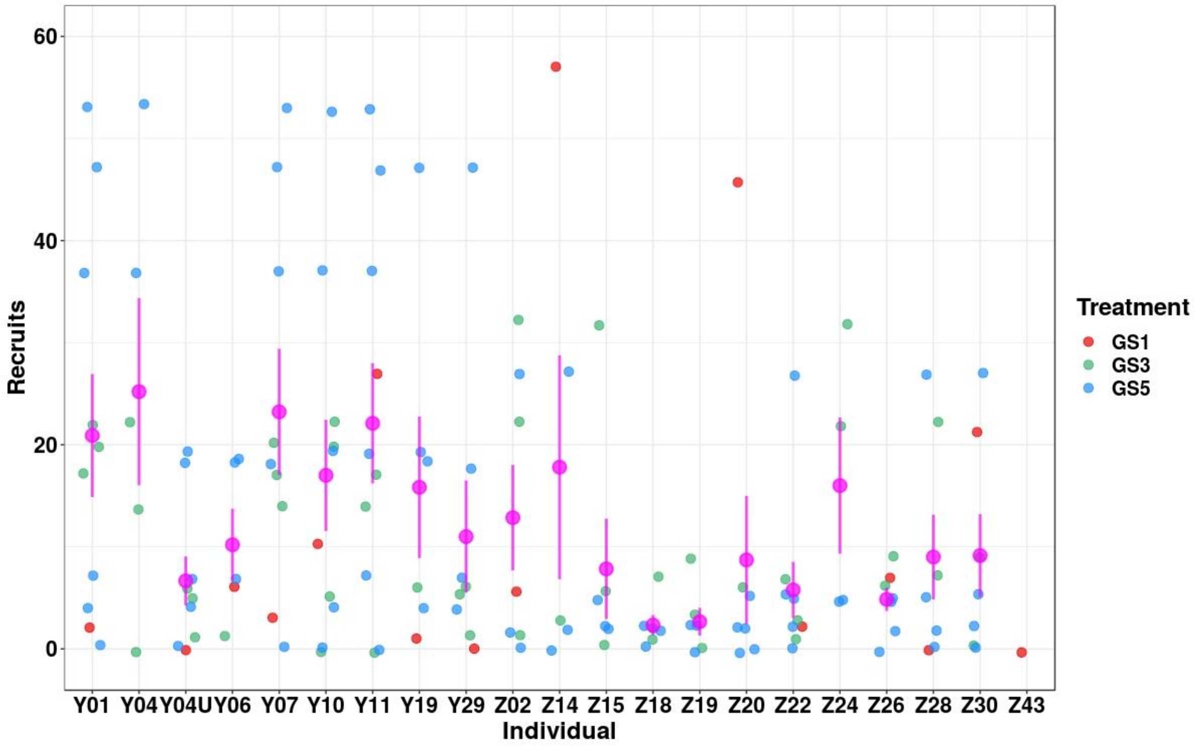

Figure S3: Plot displaying the number of birds that recruited to playbacks containing the calls of each individual male whose calls were used in the study. Playbacks where the male was the lone caller (GS1) are displayed in red, playbacks where his calls contributed to a playback containing 3 males in total (GS3) are in green, and those where he contributed to a playback containing a total of five males (GS5) are in blue. The mean number for each male is denoted by the magenta point, with the error bars representing the standard error of the mean. Note that each red dot is a unique trial, while trials denoted by a green dot are replicated 3 times in the graph, and those that are blue are replicated 5 times.

Supplementary discussion

Order and date effects

The best supported model (*model 3*; Tables 1 and 2) contained a negative effect of trial number (Table 2). This is suggestive of a habituation effect, whereby recruitment tended to decline as the number of trials carried out at each location increased.

We also found some support for a weak effect of date on recruitment. Date was not present in the top model (*model 3*), but did feature in *model 2*, within the top set of models (see Table 1). This indicates that fewer birds were recruited as the breeding season progressed, perhaps reflecting changes in parental investment, although collinearity between date and trial number renders interpretation difficult. It is important to note that the effect of date was not robust once the quasi-correction to model outputs had been applied (GLMM, b (s.e.) = -0.022 (0.014), z = -1.54, p = 0.12).

References

1. Woods, RD. 2016 Collective responses to acoustic threat information in jackdaws. PhD Thesis, University of Exeter

2. Woods RD, Kings M, McIvor GE, Thornton A. 2018 Caller characteristics influence recruitment to collective anti-predator events in jackdaws. *Sci Rep*. **8**, 7343. (doi:10.1038/s41598-018-25793-y)
